# Citywide community-based colorectal cancer screening in urban Shanghai: age and sex variations in risk mitigation from 1973 to 2020

**DOI:** 10.3389/fonc.2025.1707133

**Published:** 2025-12-09

**Authors:** Mengyin Wu, Yangming Gong, Chunxiao Wu, Peng Peng, Lei Chen, Qi Li, Liang Shi, Yongmei Xiang, Jianming Dou, Yi Pang, Xiaocong Zhang, Kai Gu, Yan Shi

**Affiliations:** 1 Division of Noncommunicable Diseases and Injury, Shanghai Municipal Center for Disease Control and Prevention, Shanghai, China; 2 Division of Public Health Informatics, Shanghai Municipal Center for Disease Control and Prevention, Shanghai, China

**Keywords:** colorectal cancer, screening, trend, interrupted time-series analysis, epidemiological study

## Abstract

**Aims:**

To evaluate the impact of Shanghai’s community colorectal cancer (CRC) screening program on CRC epidemiology and risk reduction.

**Materials and methods:**

Data from the Shanghai Cancer Registry (1973-2020) were analyzed. Launched in 2013, the program targets residents aged 50-74, offering free risk assessment (questionnaire and fecal tests). High-risk individuals are advised for colonoscopy (non-mandatory). Interrupted time-series analysis assessed age-standardized incidence and mortality trends.

**Results:**

Over 3 million individuals were screened, with 18.7% high-risk and 27-32% undergoing colonoscopy. Screening did not significantly reduce CRC incidence but led to a mortality decline (annual percent change [APC]: 1.07% to −3.16%, *P* < 0.001). Females showed greater benefit, with incidence APC dropping from 1.93% to −0.28% (*P* < 0.001). No incidence reduction was seen in younger or older groups, with under-50s showing rising incidence. However, elderly mortality decreased significantly (APC: 1.47% to −3.03%, *P* < 0.001).

**Conclusion:**

The program significantly reduced CRC mortality, particularly in females and the elderly, though incidence trends varied by age. Despite a significant reduction in mortality, the observed stability in overall CRC incidence is an expected finding in the initial years of a screening program. This pattern is largely due to increased detection of prevalent pre-existing cases during the first screening round, which may temporarily offset the decline in incident cases.

## Introduction

Colorectal cancer (CRC) is one of the most diagnosed cancer worldwide, imposing a substantial disease burden on public health due to its high incidence. According to the latest estimates from GLOBOCAN 2022, there were nearly 2 million new cases and 1 million deaths of CRC in 2022, corresponding to the 3^rd^ in incidence spectrum of malignancies and the 2^nd^ in mortality spectrum, respectively (1). Over the past few decades, the incidence and mortality of CRC has decreased due to effective screening measures, early interventions and better treatment options in some areas (2–4). However, more recent data suggested that fewer residents were up to date with screening and the risk of CRC is trending younger, leading to concern that rates might be plateauing (5).

Fecal occult blood tests (FOBT), fecal immunochemical testing (FIT) and colonoscopy are the most common screening methods worldwide, but the impact of screening program remain largely indefinite (6, 7). Each screening method has its advantages and limitations, and the individual characteristics of each method also influence patient and physician perceptions and preferences, which in turn could influence the informed decision-making process for selecting an appropriate screening method. Applying multiple screening options may help increase population acceptance of screening, but data on the effect of population-based organized screening programs on CRC incidence and mortality are insufficient (8, 9).

In this study, we collected the data on CRC from 1973–2020 in urban Shanghai and performed an interrupted time-series analysis to estimate the effect of community CRC screening program on the incidence and mortality of CRC, to clarify the potential role of screening in reducing the risk of CRC, extending the survival of CRC patients, and provide a realistic foundation for CRC prevention and treatment.

## Materials and methods

### Data sources

The case data on CRC in urban Shanghai from 1973–2020 were obtained through the Shanghai Cancer Registry (SCR), an associate member of the International Association of Cancer Registries (IACR), and the Vital Statistics System of Shanghai Municipal Center for Disease Control and Prevention (Shanghai CDC), as previously described (10, 11). Briefly, the SCR has formed a standard system to collect, process, and report incident cancer cases in Shanghai, and complete incidence data have been available since 1973. The corresponding population data of urban Shanghai were obtained from the Shanghai Public Security Bureau, and the world standard million population (Segi 1960) was used to calculate the age-standardized rates of incidence and mortality (12).

### Community CRC screening program in Shanghai

Shanghai, a megacity on China’s east coast, covers 6,340.5 km² across 16 districts and had over 24 million residents by 2020. In 2008, the Shanghai CDC piloted a CRC screening program in Qibao, Minhang District. By 2012, the Shanghai Municipal Government designated CRC screening as a key public health initiative, officially launching it citywide in 2013. The program, implemented through over 200 community health centers across all 16 districts, offers free CRC screening to residents aged 50-74 (those ≥75 may participate voluntarily). Its goal is to enhance population health through early detection.

**Figure 1:**
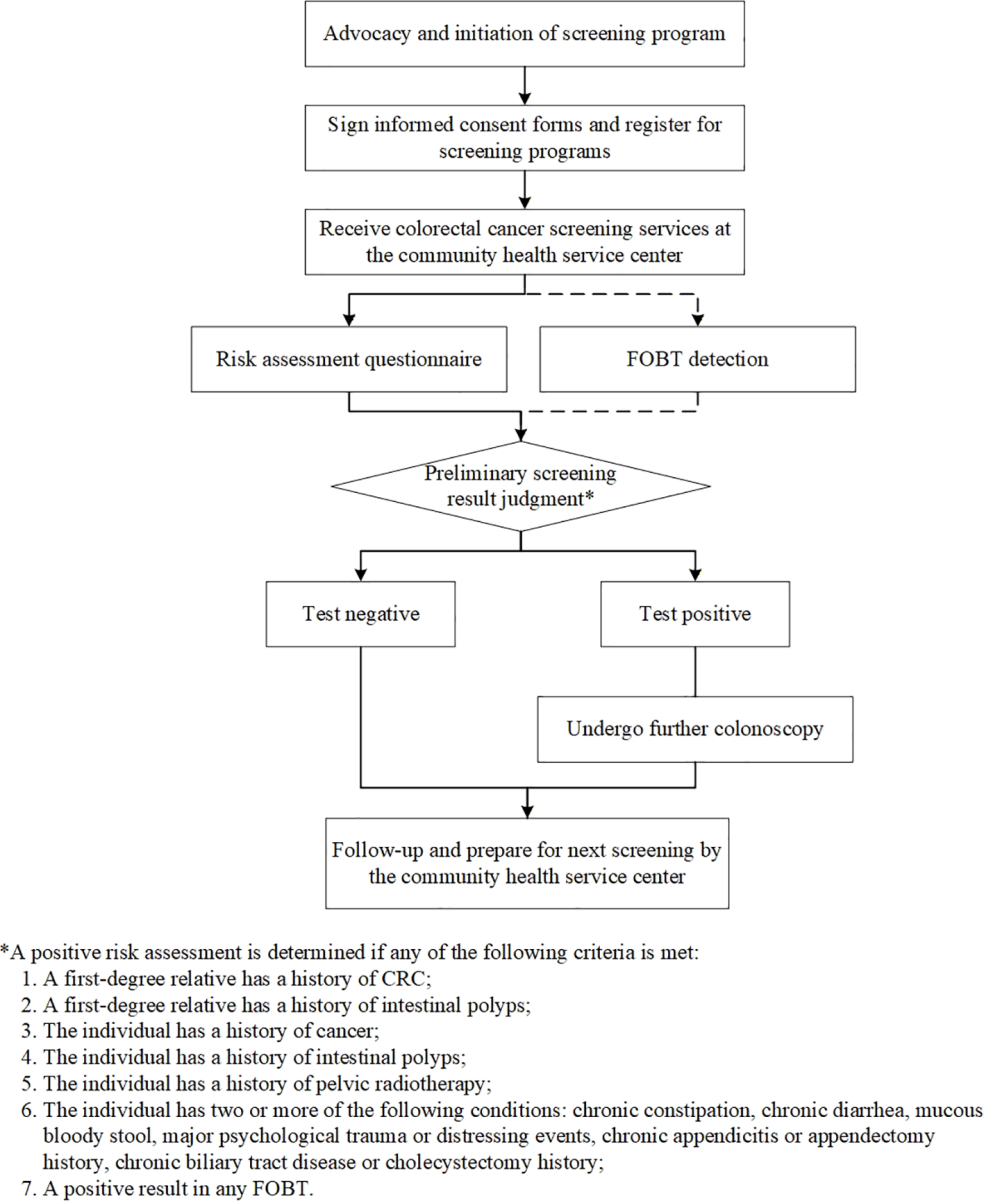
Flowchart of the citywide community-based colorectal cancer screening process in Shanghai.

The community CRC screening process is shown in **Figure 1**. After registration and signing informed consent, residents receive screening services at community health centers, which include a risk assessment questionnaire and two qualitative fecal immunochemical tests (FITs) conducted at least 7 days apart. The risk assessment collects demographic information (age, sex, marital status, education, occupation), along with medical history (chronic gastrointestinal diseases, colorectal polyps, cancer), lifestyle factors (smoking), and family history of CRC. Residents who are comprehensively assessed as at high risk of CRC are referred to designated secondary-level or higher hospitals for colonoscopy, which must reach the ileocecal valve and is performed according to national standards. A 5% random audit of all colonoscopy and pathology reports was implemented. Reports were selected from the central information system using a computer-generated random number sequence. Specialized staff evaluated these reports for completeness, verified key quality indicators (including cecal intubation rates), and assessed the consistency between endoscopic findings and pathological diagnoses. Based on examination findings, patients receive appropriate treatment. Negative cases are incorporated into community health management and reassessed the following year. All colonoscopy results and pathological findings are recorded in standardized forms and reported through the system.

### Statistical analysis

Interrupted time-series analysis was performed to estimate the effects of community CRC screening program on the age-standardized incidence and mortality rates of CRC. The model constructed in this study could be expressed as follow:


$$Y=b_{0}+b_{1}\times T+b_{2}\times D+b_{3}\times P+e$$

Where *Y* was a dependent variable representing the age-standardized rates. *T*, *D* and *P* were considered as independent variables. *T* referred to the years passed from the start of the observational period; *D* referred to observation collected before (=0) or after (=1) the screening implementation; *P* referred to time passed since the screening has started (before screening implementation has started *P* was equal to 0). $b_{0}$ was the level of explained variables at the beginning. $b_{1}$ estimated the baseline trend before the screening implementation. $b_{2}$ estimated the effect of the screening on the change of age-standardized rates. $b_{3}$ reflected the changed trend after the screening implementation (the change in the slope). *e* was the error term.

Besides, subgroup analyses by sex and age were conducted to identify the potential sources of heterogeneity and access the consistency of screening effects in different population on the observed rates of incidence and mortality. Based on the age range of the target population of the screening program, we included people under 50 years old as the younger group, and people 50 years and older as the older group to observe the effect of the screening program. To better assess the effectiveness of the screening program, we conducted further analysis to examine the trends in age-standardized incidence and mortality rates of CRC before and after the initiation of the screening program in different age subgroups (aged 50–59 *vs*. 60–74 *vs*. 75-). All statistical analyses were conducted using STATA 17.0 (STATA Corp., College Station, TX, USA), and statistical significance was attributed to two-sided *P*-values < 0.05.

## Results

Shanghai’s citywide CRC screening program, launched in 2013, has provided over 6 million screenings to more than 3 million residents, covering 38.40% of the eligible 50–74 age group. The program has achieved good participation rates, with 70% of residents completing the recommended two FIT tests within 7 days and 90% completing both tests within a month, averaging 1.4-1.8 tests per participant. Screening results show that 10% tested positive on at least one FIT, while 9% were identified as high-risk based on family history and other key criteria, leading to an overall 18.7% high-risk determination through combined questionnaire and FIT assessment. Among these high-risk individuals, 27%-32% underwent follow-up colonoscopy, which currently detects new CRC cases in 5%-7% of those examined.

### Overall analysis

During the nearly 50-year study period, urban Shanghai recorded 119,746 new CRC cases (53.50% male, 46.50% female) and 66,745 CRC deaths (53.56% male, 46.44% female). Diagnosis staging data for 2002–2020 cases are provided in **Supplementary Table S1**. With socioeconomic development and population aging, Shanghai’s CRC risk showed a fluctuating upward trend. Age-standardized incidence rose from 12.44/100,000 in 1973 to 26.82/100,000 in 2020 (AAGR: 2.46%), while mortality increased from 7.94/100,000 to 10.84/100,000 (AAGR: 0.78%).

**Figure 2:**
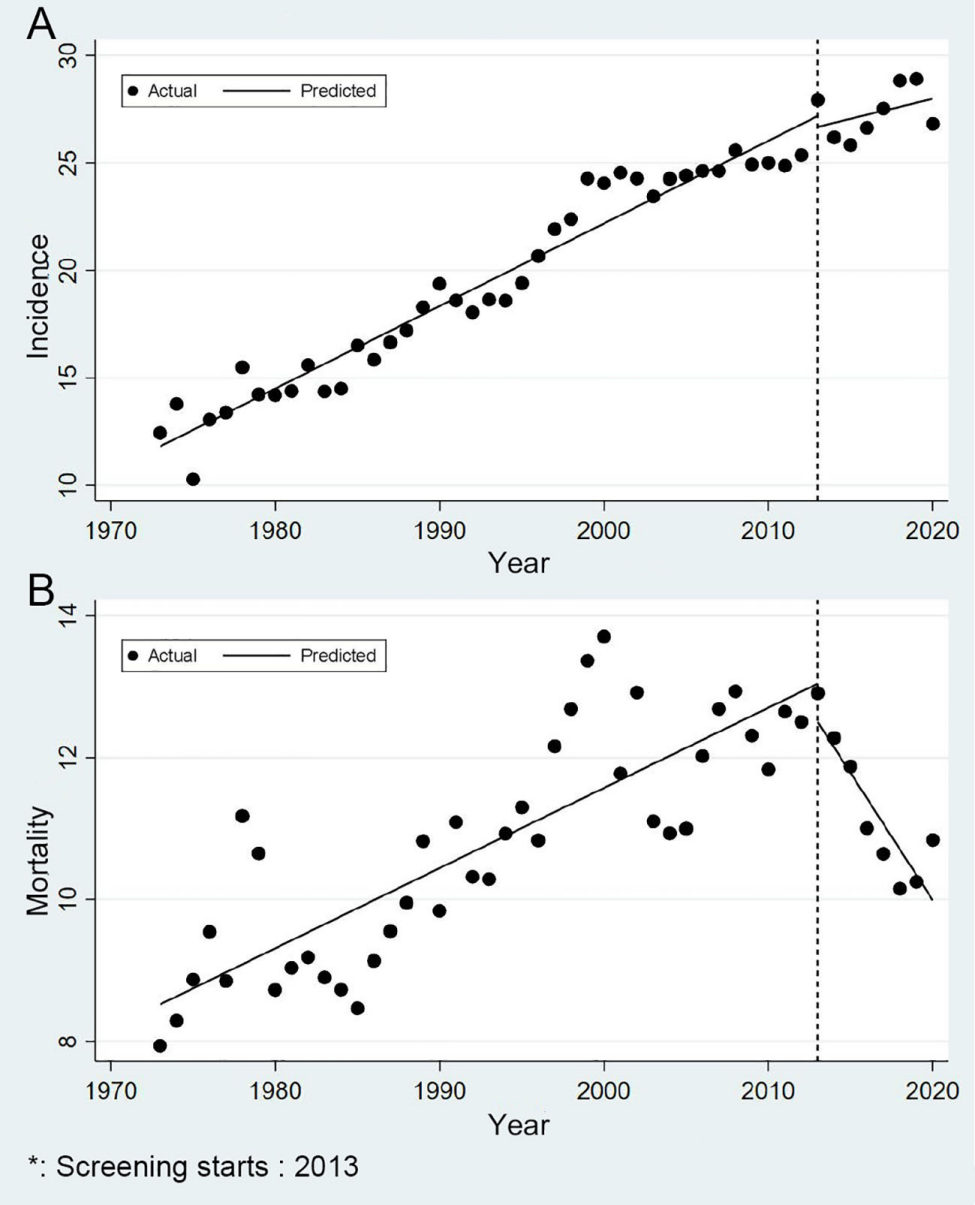
Trends in incidence and mortality of colorectal cancer in urban Shanghai, 1973-2020 (**A** for the age-standardized incidence rates and **B** for the age-standardized mortality rates).

The screening program showed greater impact on mortality reduction than incidence reduction (**Figure 2**). Pre-screening, CRC incidence showed an APC of 2.11%, which decreased to 0.68% post-implementation (*P* = 0.230). Mortality trends changed more significantly: pre-screening APC was 1.07%, declining to -3.16% post-screening (*P* < 0.001), indicating a substantial mortality reduction (**Figure 2B**).

### Subgroup analysis

**Figure 3:**
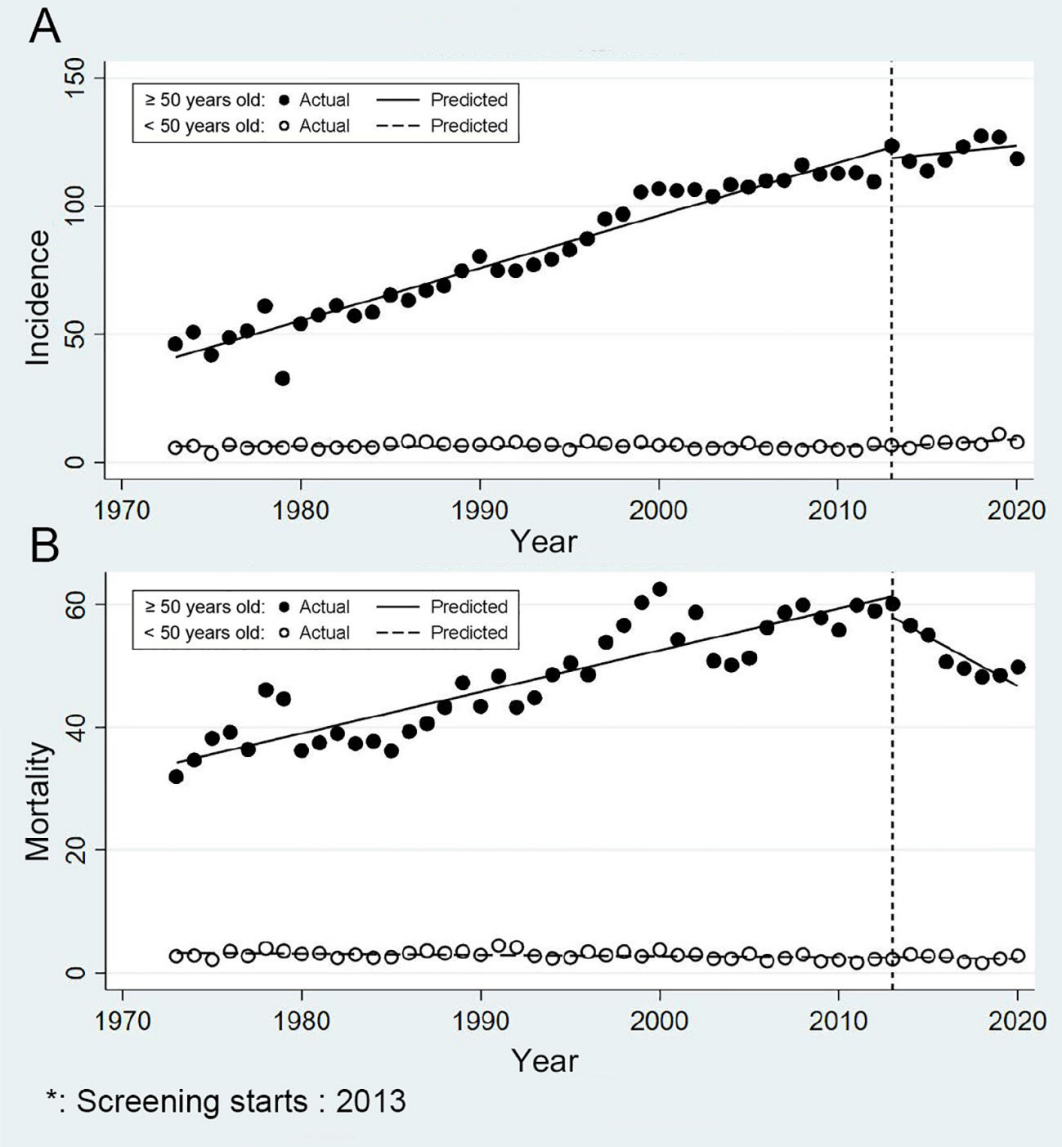
Age-specific trends in incidence and mortality of colorectal cancer in urban Shanghai, 1973-2020 (**A** for the age-standardized incidence rates and **B** for the age-standardized mortality rates).

**Figure 3** showed the trends in CRC risk among urban Shanghai residents from 1973 to 2020 across various age groups. The age-standardized incidence and mortality of CRC were substantially higher among individuals aged 50 years and older compared to those under 50 years old. Among the elderly (50 years and above), the age-standardized incidence rose from 46.07/100,000 in 1973 to 118.54/100,000 in 2020. Meanwhile, among the younger population (under 50 years), the incidence increased from 5.56/100,000 in 1973 to 7.78/100,000 in 2020. In terms of mortality, the age-standardized rate among the elderly increased from 31.93/100,000 in 1973 to 49.80/100,000 in 2020, whereas among the younger population, it remained stable, ranging from 2.68/100,000 in 1973 to 2.75/100,000 in 2020.

The current screening did not demonstrate an effect in reducing the incidence risk of CRC in either younger or older populations (**Figure 3A**). Prior to the screening program’s introduction, the age-standardized incidence APC among the younger was a slight decrease of -0.03%, but afterward, it even notably surged to 5.50% (*P* = 0.003). In older populations, the age-standardized incidence APC dropped from 2.79% prior to the screening program to 0.56% following its implementation (*P* = 0.050). During the entire study period, there was no significant change in age-standardized mortality among the younger. Conversely, among the elderly, the CRC mortality risk showed a significant decrease, with the age-standardized mortality APC decreasing from 1.47% to -3.03% (*P* < 0.001) (**Figure 3B**). Further results for subgroup analyses by age are presented in **Supplementary Figures S1** and **S2**.

**Figure 4:**
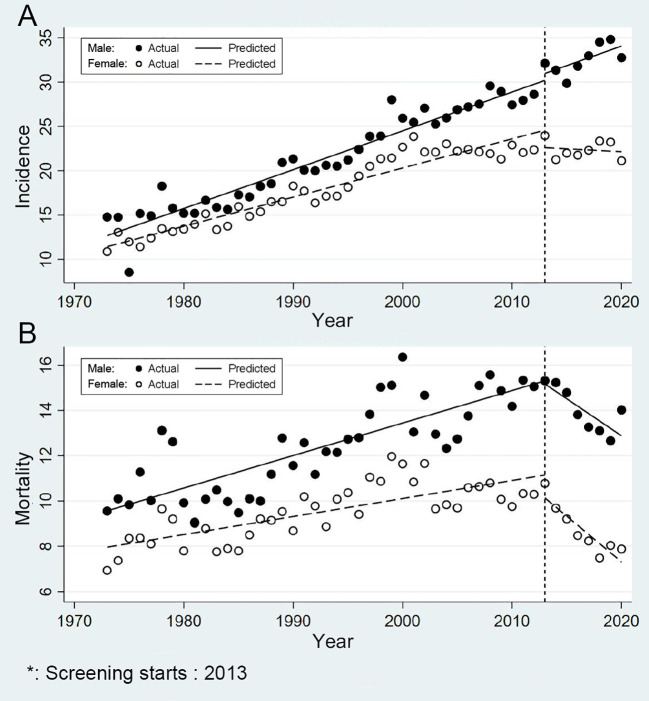
Sex-specific trends in incidence and mortality of colorectal cancer in urban Shanghai, 1973-2020 (**A** for the age-standardized incidence rates and **B** for the age-standardized mortality rates).

The trends of incidence and mortality of CRC in urban Shanghai from 1973 to 2020 in different sex groups are shown in **Figure 4**. Briefly, the age-standardized incidence and mortality rates were slightly higher in males than in females, and the changes in the trends of incidence and mortality in different sex groups exhibited various characteristics. Among males, the age-standardized incidence rose from 14.78/100,000 in 1973 to 32.76/100,000 in 2020. Meanwhile, among females, the incidence increased from 10.87/100,000 in 1973 to 21.12/100,000 in 2020. In terms of mortality, the age-standardized rate among males increased from 9.55/100,000 in 1973 to 14.02/100,000 in 2020, whereas among females, it remained stable, ranging from 6.93/100,000 in 1973 to 7.88/100,000 in 2020.

In males, the effect of screening on reducing the incidence risk of CRC is currently not significant (**Figure 4A**). Before the CRC screening program, the age-standardized incidence APC among males was 2.20% and post-screening, the APC decreased to 1.37%, but this change was not statistically significant (*P* = 0.987). In contrast, among females, the incidence risk of CRC significantly decreased after the screening program was launched. The APC dropped from 1.93% prior to the screening program to -0.28% following its implementation (*P* = 0.013). And consistent with the results of the primary analysis, the effect of screening on reducing the mortality risk of CRC was significant in both sexes (**Figure 4B**). Specifically, for males, the APC decreased from 1.18% to -2.31% (*P* < 0.001), while for females, it declined from 0.85% to -4.57% (*P* < 0.001), highlighting the program’s potential in reducing CRC deaths.

## Discussion

This study estimated the effects of community screening on the risk of CRC in urban Shanghai from 1973-2020. The results indicated a significant reduction in the mortality risk of CRC among residents who underwent screening, and that the effects of screening varied widely across age and sex groups. However, the impact on lowering the incidence of CRC remained inconclusive.

Previous studies have demonstrated that the progression and advancement of CRC follow a sequential process, involving complex interactions among various factors and stages (13). Most CRC begin with an aberrant crypt, evolving into a neoplastic precursor lesion (polyp), and ultimately progress to CRC (14). This evolutionary process is estimated to take a 10-15-year period, thus becoming a key window for the early prevention and treatment of CRC. Apart from non-modifiable risk factors such as age, race, genetics and family history, the development of CRC is also tightly associated with several modifiable risk factors, including cigarette smoking, alcohol consumption, diet, obesity, physical activity. Notably, this prolonged preclinical phase underpins the rationale for population-based screening. It allows for the interception of lesions at early, treatable stages before they progress to invasive cancer. Consistent with this, our diagnostic staging data (**Supplementary Table S1**), which showed a trend towards earlier-stage diagnosis, suggested that the screening program contributed to mortality reduction primarily by enabling earlier treatment. This, combined with the opportunity during screening to counsel participants on modifying the lifestyle risks mentioned above, underscores the program’s dual role in primary and secondary prevention.

Screening is an effective measure for cancer prevention and control in the population. Despite the increasing risk of CRC worldwide over the past few decades, the incidence and mortality rates have stabilized or declined in some developed countries with high morbidity (15, 16). This improvement can be attributed to the implementation of appropriate screening measures and the adoption of best practices in CRC treatment and management (17–21). The program’s launch coincided with the maturation of minimally invasive surgical techniques in China—particularly the widespread adoption of laparoscopy after 2010 and refined procedures such as Natural orifice specimen extraction surgery (NOSES) around 2013. While these therapeutic advances contributed to the secular mortality reduction trend, the ITS analysis demonstrates a significant post-screening shift in the mortality trend slope. The screening program systematically identified early-stage cases, enabling full realization of modern surgical benefits. The accelerated mortality declines thus likely stems from combined systematic early detection via screening and optimal treatment with advanced surgical care.

Previous large cohort studies have demonstrated the ability of colonoscopy to detect precancerous lesions through direct visualization (22, 23). The sensitivity of colonoscopy for detecting CRC is over 95%, and which for detecting advanced adenomas is 88%-98% (7, 24, 25). Several case-control studies have shown that colonoscopy could reduce the incidence of CRC by 53%-72% and related mortality by 31% (26, 27). However, evidence for the reduction in randomized controlled trials and some trials directly comparing outcomes of colonoscopy with other screening tests is inconsistent (28–30). Furthermore, the quality of evidence varies among different screening methods due to differences in price, convenience of the procedure, invasiveness, and acceptance. In recent years, the level of diagnosis and treatment of CRC in China has risen a step. Endoscopic technology has been continuously developed, and the treatment process causes less trauma, has fewer complications, and results in faster recovery. The whole treatment system is also more precise and personalized. At the same time, new drugs for CRC have been coming out since 2000, providing patients with more options. It must be acknowledged that advances in treatment have contributed to the reduction of the risk of death from CRC, but early detection of CRC cases through screening is the premise and foundation of all this.

Since the late 1990s and early 2000s, screening based on stool or endoscopic options has been introduced as a measure of prevention and treatment of CRC in the US (31–34). The incidence of CRC might initially increase after the introduction of screening methods because of the detection of some undiagnosed diseases, while in the long term, the incidence and mortality of CRC might gradually decrease due to the removal of precancerous polyps during colonoscopy (35). Similar trends have also been observed in Japan and Israel, two other countries that have adopted screening measures and established early detection programs since the 1990s (36, 37). However, considering that the development of CRC is influenced by a combination of environmental and genetic factors, the extent to which screening interventions could reduce the incidence and mortality of CRC is still difficult to determine. In Shanghai, CRC screening is mainly conducted through community organizations. Due to economic considerations, the main screening method is a combination of risk assessment through questionnaire and FOBT detection and further colonoscopy is recommended for high-risk population.

The study reveals that while Shanghai’s community CRC screening program hasn’t significantly reduced age-standardized incidence in the short term, it has markedly decreased age-standardized mortality. The limited acceptance of colonoscopy due to its invasiveness and cost may explain this pattern, as primarily symptomatic individuals and those with family history comply with screening, leading to earlier detection and treatment that reduces mortality more than incidence. Notably, our program’s colonoscopy uptake rate of 27-32% is comparable to, and in some cases exceeds, that reported in other community-based screening initiatives facing similar barriers (38). This comparison suggests that the observed mortality reduction provides a credible estimate of the program’s real-world effectiveness, rather than an idealized efficacy, and underscores the significant achievement in motivating participation within a challenging context. Notable age and gender disparities emerged in screening effectiveness. The increased incidence of CRC in the under-50 population is attributable to multiple factors. In addition to global trends and diagnostic advances, the affluent, health-conscious context in Shanghai is identified as a key contributor. Widespread screening publicity is postulated to have spurred voluntary check-ups among younger adults, generating an “awareness spillover effect” that enhanced case detection in this non-targeted group. For individuals over 50 years old, the age-standardized incidence showed a short-term increase immediately following the start of screening, but the long-term growth rate decelerated. This could be explained by the early detection of some potential cases among those who participated in the screening programs, as screening ultimately reduces the risk of developing CRC in the long run. The mortality risk among individuals over 50 years old significantly decreased after the initiation of screening programs. In contrast, those aged 0–49 may not have benefited from the screening programs due to their lack of participation. It is noteworthy that, following the initiation of screening programs, the risk of CRC incidence significantly decreased among females, whereas no such reduction was observed among males. Additionally, the extent of reduction in mortality risk from CRC was greater among females compared to males. This discrepancy may be attributed to higher compliance rates among females towards screening programs, as well as differences in lifestyle habits between the two genders.

This study’s strength lies in being the first comprehensive evaluation of Shanghai’s community CRC screening program and its impact on CRC risk reduction from 1973-2020. As Shanghai’s rapid economic development has significantly altered population demographics and lifestyle factors, particularly diet, its CRC incidence and mortality trends mirror early patterns observed in Western developed nations, making these findings valuable for other Asian regions undergoing similar transitions. The study benefits from reliable data sources and a representative population. However, several limitations should be noted. First, the analysis focused on relatively simple screening methods without comparing multiple approaches. Second, as an observational study limited to Shanghai, the long-term effects of screening require further monitoring. Third, while analyzing population-level incidence and mortality data, the study lacked adjustments for CRC risk factors. It is also important to acknowledge concurrent improvements in CRC regimens in Shanghai, such as the increased adoption of laparoscopic surgery and refined chemotherapy protocols, which likely contributed to this trend (39, 40). Continued long-term observation and additional research are needed to fully understand screening’s impact on CRC risk reduction.

## Conclusion

In summary, Shanghai’s community-based CRC screening program was associated with a significant reduction in CRC mortality, particularly among the elderly and females—with the latter also showing stabilized incidence rates. This dissociation underscores that the program’s primary benefit was achieved through stage shift via early detection, rather than a decrease in overall incidence. These findings confirm the program’s vital role in alleviating the CRC disease burden and emphasize the importance of early detection for improving survival.

## Data Availability

The raw data supporting the conclusions of this article will be made available by the authors, without undue reservation.
