# Supplementary material for: Citywide community-based colorectal cancer screening in urban Shanghai: age and sex variations in risk mitigation from 1973 to 2020
Source: Front Oncol. 2025 Dec 9;15:1707133. doi: 10.3389/fonc.2025.1707133 (PMC12722893; doi:10.3389/fonc.2025.1707133)
Supplement: Supplementary file 1 [file DataSheet1.docx]

**Citywide Community-Based Colorectal Cancer Screening in Urban Shanghai: Age and Sex Variations in Risk Mitigation from 1973 to 2020**

**Supplementary Material**

Table S1. Staging of CRC in Urban Shanghai from 2002 to 2020

Figure S1. Trends in age-stratified incidence rates of CRC in urban Shanghai from 1973-2020 (A for the age-standardized incidence rates of the 50-59 age group, B for the 60-74 age group and C for the 75- age group)

Figure S2. Trends in age-stratified mortality rates of CRC in urban Shanghai from 1973-2020 (A for the age-standardized mortality rates of the 50-59 age group, B for the 60-74 age group and C for the 75- age group)

**Table S1. Staging of CRC in Urban Shanghai from 2002 to 2020**

| **Year** | **Cases** | **Cases with definite staging (%)** | **Stage I**  **(%)** | **Stage II**  **(%)** | **Stage III**  **(%)** | **Stage IV**  **(%)** |
| --- | --- | --- | --- | --- | --- | --- |
| **2002** | 2861 | 1521 (53.2%) | 169 (11.1%) | 554 (36.4%) | 405 (26.6%) | 393 (25.8%) |
| **2003** | 2862 | 1371 (47.9%) | 153 (11.2%) | 502 (36.6%) | 347 (25.3%) | 369 (26.9%) |
| **2004** | 3066 | 1473 (48.0%) | 168 (11.4%) | 563 (38.2%) | 381 (25.9%) | 361 (24.5%) |
| **2005** | 3103 | 1592 (51.3%) | 154 (9.7%) | 598 (37.6%) | 445 (28.0%) | 395 (24.8%) |
| **2006** | 3249 | 1671 (51.4%) | 208 (12.4%) | 599 (35.8%) | 482 (28.8%) | 382 (22.9%) |
| **2007** | 3342 | 1772 (53.0%) | 184 (10.4%) | 620 (35.0%) | 543 (30.6%) | 425 (24.0%) |
| **2008** | 3565 | 1932 (54.2%) | 234 (12.1%) | 639 (33.1%) | 560 (29.0%) | 499 (25.8%) |
| **2009** | 3601 | 1994 (55.4%) | 239 (12.0%) | 737 (37.0%) | 561 (28.1%) | 457 (22.9%) |
| **2010** | 3705 | 1956 (52.8%) | 197 (10.1%) | 697 (35.6%) | 600 (30.7%) | 462 (23.6%) |
| **2011** | 3789 | 1985 (52.4%) | 198 (10.0%) | 665 (33.5%) | 644 (32.4%) | 478 (24.1%) |
| **2012** | 3892 | 2141 (55.0%) | 273 (12.8%) | 711 (33.2%) | 633 (29.6%) | 524 (24.5%) |
| **2013** | 4382 | 2281 (52.1%) | 352 (15.4%) | 780 (34.2%) | 648 (28.4%) | 501 (22.0%) |
| **2014** | 4256 | 2234 (52.5%) | 426 (19.1%) | 677 (30.3%) | 568 (25.4%) | 563 (25.2%) |
| **2015** | 4270 | 2162 (50.6%) | 370 (17.1%) | 668 (30.9%) | 544 (25.2%) | 580 (26.8%) |
| **2016** | 4430 | 1961 (44.3%) | 324 (16.5%) | 548 (27.9%) | 567 (28.9%) | 522 (26.6%) |
| **2017** | 4706 | 2173 (46.2%) | 323 (14.9%) | 595 (27.4%) | 613 (28.2%) | 642 (29.5%) |
| **2018** | 4969 | 2160 (43.5%) | 290 (13.4%) | 618 (28.6%) | 593 (27.5%) | 659 (30.5%) |
| **2019** | 5134 | 2210 (43.0%) | 316 (14.3%) | 631 (28.6%) | 628 (28.4%) | 635 (28.7%) |
| **2020** | 4813 | 1986 (41.3%) | 283 (14.2%) | 602 (30.3%) | 569 (28.7%) | 532 (26.8%) |


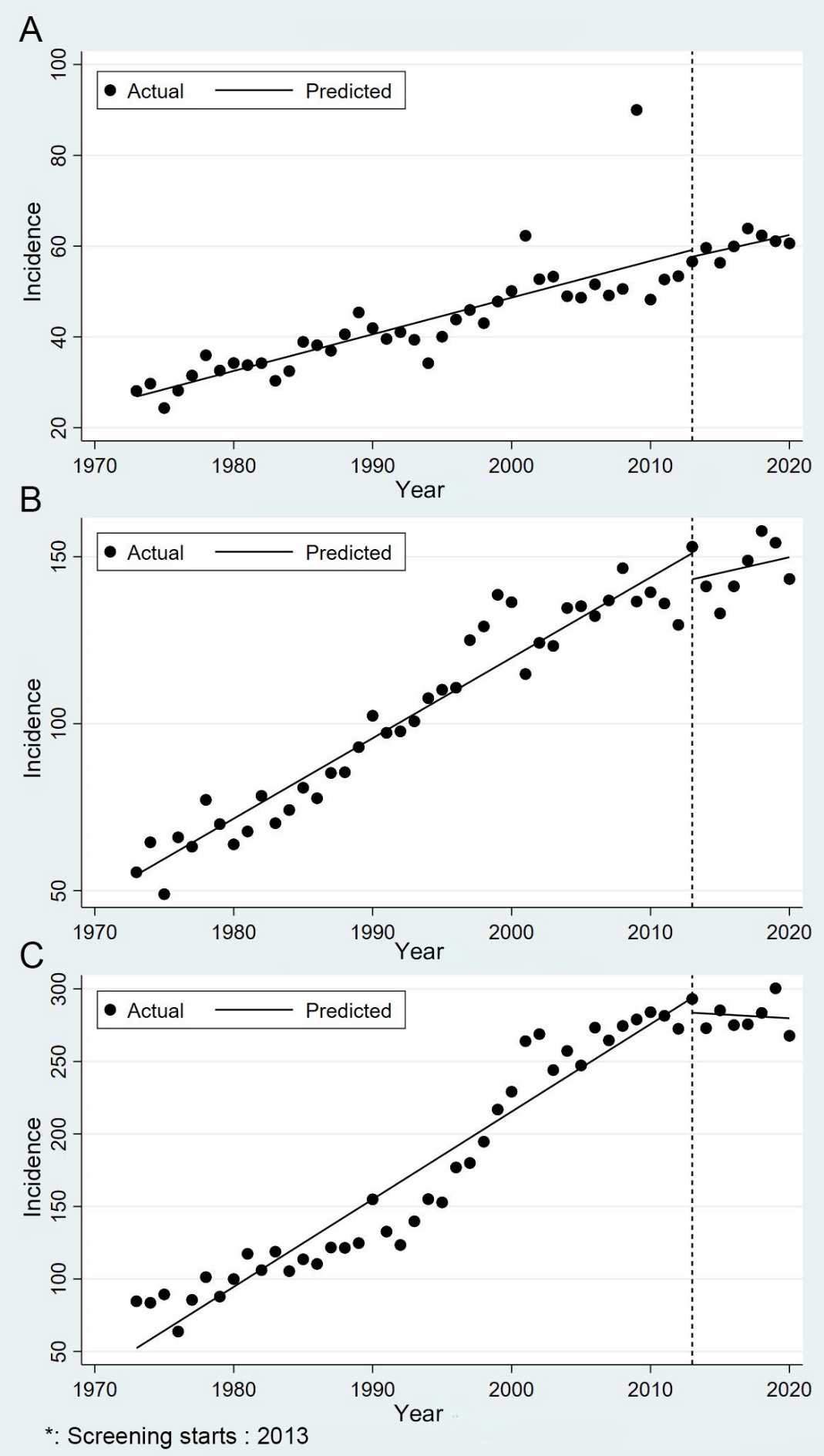


**Figure S1. Trends in age-stratified incidence rates of CRC in urban Shanghai from 1973-2020 (A for the age-standardized incidence rates of the 50-59 age group, B for the 60-74 age group and C for the 75- age group)**


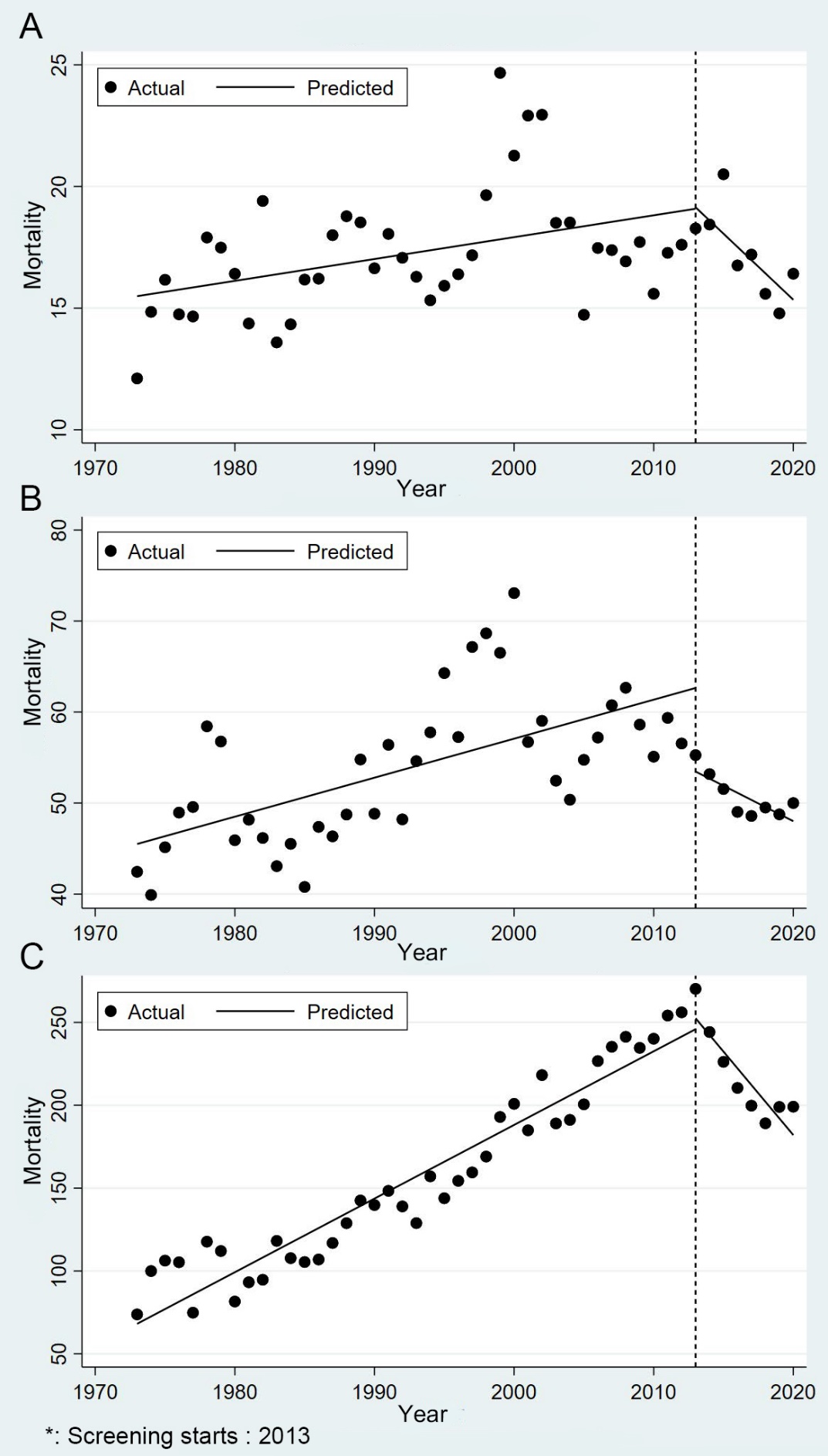


**Figure S2. Trends in age-stratified mortality rates of CRC in urban Shanghai from 1973-2020 (A for the age-standardized mortality rates of the 50-59 age group, B for the 60-74 age group and C for the 75- age group)**
